# Beer Phenolic Composition of Simple Phenols, Prenylated Flavonoids and Alkylresorcinols

**DOI:** 10.3390/molecules25112582

**Published:** 2020-06-02

**Authors:** Anna Boronat, Natalia Soldevila-Domenech, Jose Rodríguez-Morató, Miriam Martínez-Huélamo, Rosa M. Lamuela-Raventós, Rafael de la Torre

**Affiliations:** 1 Integrative Pharmacology and Systems Neuroscience Research Group, Neurosciences Research Program, IMIM-Institut Hospital del Mar d’Investigacions Mèdiques, Dr. Aiguader 88, 08003 Barcelona, Spain; aboronat@imim.es (A.B.); nsoldevila@imim.es (N.S.-D.); jrodriguez1@imim.es (J.R.-M.); 2 Department of Experimental and Health Sciences, Universitat Pompeu Fabra (CEXS-UPF), Dr. Aiguader 80, 08003 Barcelona, Spain; 3 Medtep Inc., 08025 Barcelona, Spain; 4 Spanish Biomedical Research Centre in Physiopathology of Obesity and Nutrition (CIBERObn), Instituto de Salud Carlos III (ISCIII), 28029 Madrid, Spain; lamuela@ub.edu; 5 Department of Nutrition, Food Science and Gastronomy, School of Pharmacy and Food Sciences and XaRTA, Institute of Nutrition and Food Safety (INSA-UB), University of Barcelona, 08921 Santa Coloma de Gramanet, Spain; mmartinezh@ub.edu

**Keywords:** beer, antioxidants, prenylated flavonoids, tyrosol, hydroxytyrosol, alkylresorcinols

## Abstract

Beer is a fermented beverage with beneficial phenolic compounds and is widely consumed worldwide. The current study aimed to describe the content of three families of phenolic compounds with relevant biological activities: prenylated flavonoids (from hops), simple phenolic alcohols (from fermentation) and alkylresorcinols (from cereals) in a large sample of beers (*n* = 45). The prenylated flavonoids analyzed were xanthohumol, isoxanthohumol, 6- and 8-prenylnaringenin. The total prenylated flavonoids present in beer ranged from 0.0 to 9.5 mg/L. The simple phenolic alcohols analyzed were tyrosol and hydroxytyrosol, ranging from 0.2 to 44.4 and 0.0 to 0.1 mg/L, respectively. Our study describes, for the first time, the presence of low amounts of alkylresorcinols in beer, in concentrations ranging from 0.02 to 11.0 µg/L. The results in non-alcoholic beer and the differences observed in the phenolic composition among different beer types and styles highlight the importance of the starting materials and the brewing process (especially fermentation) on the final phenolic composition of beer. In conclusion, beer represents a source of phenolic compounds in the diet that could act synergistically, triggering beneficial health effects in the context of its moderate consumption.

## 1. Introduction

Beer is a fermented alcoholic beverage containing unique kinds of phenolic compounds. Its basic ingredients are water, barley or wheat malt, hops and yeasts. Based on the type of fermentation, beer can be divided into two broad types: ale and lager. Beer has become the most prevalent form of alcohol consumption in Europe, accounting for 40% of the total alcohol consumed [1]. In general, the evidence suggests a J-shaped curve relationship between alcohol consumption and cardiovascular disease (CVD) morbidity and mortality, indicating that moderate drinkers are at lower risk than abstainers and heavy drinkers [2]. Other more specific studies observed that cardiovascular protection was only observed with moderate consumption of fermented alcoholic beverages containing phenolic compounds such as wine or beer. Nonetheless, the protective effect was not observed following moderate consumption of spirits [3,4]. In the specific case of beer, low-to-moderate consumption (up to one drink/day in women and two drinks/day in men) reduces the risk of CVD and represents no harm in relation to major chronic conditions [3,4]. Evidence suggests that beer’s beneficial health effects result from an additive effect between beer’s alcohol content and beer’s phenolic compounds [5]. Beer’s phenolic compounds derive from hops (about 30%), from barley malt (about 70%) and from the chemical transformations that these compounds undergo during the brewing process [6]. Changes in the type and proportion of each ingredient have an impact on the phenolic content which, in turn, influences the quality parameters of the resulting beer (e.g., flavor, flavor stability, color and clarity) and gives rise to different styles [7]. The total phenolic content of beer is slightly higher than in white wine and lower than in red wine [8], but it may vary according to the raw material used and brewing process parameters [7]. At the same time, its alcohol content is lower compared to other popular alcoholic drinks. Therefore, its low alcohol content together with its phenolic composition suggest beer to be a potential trigger of positive health effects while minimizing the detrimental effects associated with alcohol consumption.

An extensive variety of phenolic compounds had been described in beer including simple phenols, phenolic acids, catechins, proanthocyanins, prenylated flavonoids α- and iso-α-acids, among others [9]. To identify them, several studies have used a wide range of techniques, such as high-performance liquid chromatography (HPLC) coupled with electrochemical [10,11,12,13,14] or diode [15] array detectors, and a minority have used couplings with high resolution mass spectrometry [16]. Nevertheless, there are some gaps in the literature regarding the quantitative characterization of these compounds present in beer [7].

In terms of beer’s phenolic compounds and its potential biological activity, phenolic acids, prenylated flavonoids, α- and iso-α-acids have been the most studied. These phenolic compounds had been associated with relevant biological activities such as antioxidant, anti-inflammatory, antidiabetic and estrogenic activities [17]. However, beer can also be a source of compounds with potential toxic and pro-carcinogenic properties at higher concentrations such as carbonyl compounds and furan derivates [18].

Due to a worldwide increase in beer consumption, there is a need to characterize beer’s antioxidant profile to unveil the potential health effects attributed to moderate beer consumption. A better understanding of the phenolic composition of different types of beers is key to (i) identify the antioxidants which could be potentially responsible for the health effects attributed to moderate beer consumption and (ii) to evaluate the impact of raw material choices and brewing technology in the resulting chemical composition of beer. The aim of the present study was to explore the potential of beer as a source of antioxidant compounds in the diet, characterizing the differences between ale, lager, and non-alcoholic beers. In order to achieve this objective, we screened 45 commercially available beers for their prenylated flavonoid content, specifically those from hops (xanthohumol (XN), isoxanthohumol (IX), 6-prenylnaringenin (6PN) and 8-prenylnaringenin (8PN)), alkylresorcinols (ARs) from cereals (AR-C17:0, AR-C19:0, AR-C21:0, AR-C23:0, and AR-C25:0) and the simple phenols from tyrosine fermentation (tyrosol (TYR) and hydroxytyrosol (HT)).

## 2. Results

### 2.1. Beers Characterization

A total of 45 different beers were analyzed in the current work. The individual characteristics of analyzed beers are available in Supplementary Table S1. Beers analyzed included 18 ales, 22 lagers and five non-alcoholic beers. Within each type of beer, a further sub-classification was made in terms of their style. Information regarding alcoholic content and international bitterness units (IBU) were obtained from the manufacturer. The mean (SD) alcoholic content was 5.10 (2.15) *v*/*v* % and the mean (SD) of the IBU was 26.41 (13.11).

### 2.2. Prenylated Flavonoids

The present study analyzed the concentrations of the prenylated chalcone IX, and the prenylated flavanones XN, 8PN and 6PN. The amount of total prenylated flavonoids in the analyzed beers ranged from 0.0 to 9.47 mg/L with mean (SD) values of 0.62 (1.51) mg/L. The specific prenylated flavonoid present in largest concentrations was IX, with a mean (SD) of 0.34 (0.41) mg/L, followed by XN 0.17 (0.87) mg/L, then 6PN 0.08 (0.03) mg/L and, finally, 8PN 0.03 (0.10) mg/L. Between ale and lager beers, no statistical differences were observed in either individual or total prenylated flavonoid concentration (Table 1). However, non-alcoholic beers presented lower concentrations of IX, reaching borderline significance compared to both ale and lager (*p* = 0.06 for both) (Table 1).

### 2.3. Simple Phenols

The phenols TYR and HT were determined in all samples. The presence of TYR in beer ranged from 0.2–44.4, while HT concentration ranged from 0.0 to 0.13 mg/L. No significant differences were found in TYR levels between ale and lager (Table 1). In the case of HT, ale presented significantly greater concentrations than lager (*p* < 0.05) (0.04 (0.03) mg/L for ale and 0.02 (0.02) mg/L for lager). Regarding the phenolic content of non-alcoholic beer, TYR and HT levels were significantly lower than in ale and lager beers (*p* < 0.05 for both) (Table 1).

### 2.4. Alkylresorcinols

The present study described the presence of ARs in beer in concentrations varying from 0.02 to 11.04 µg/L. We measured a total of five ARs differing on the length of the alkyl chain, from the AR-C17:0 to AR-C25:0. The most abundant AR in all the analyzed beers was AR-C25:0 with a mean (SD) of 0.58 (1.19) µg/L (Table 1). No significant differences were found between ale, lager and non-alcoholic beers.

### 2.5. Correlation Study

**Figure 1:**
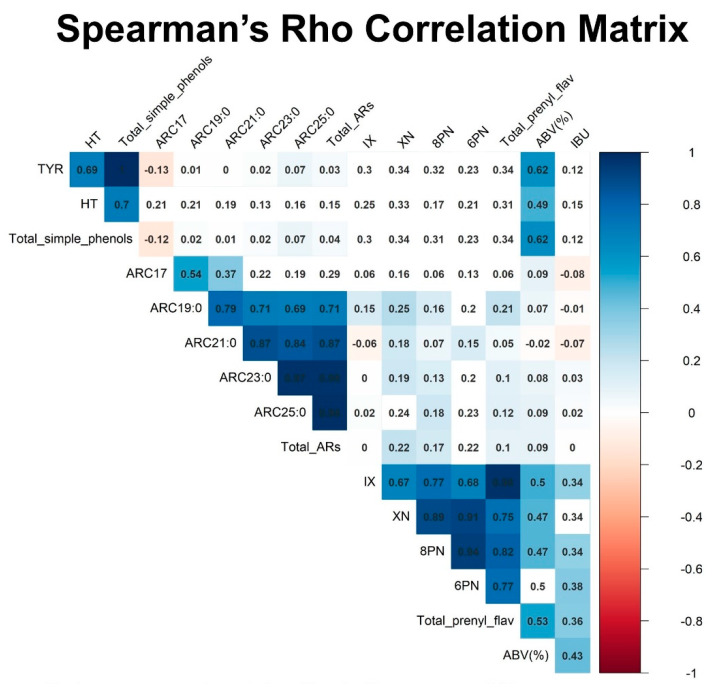
Spearman’s Rho correlation matrix of the studied phenolic compounds in the overall beer sample (*n* = 45). Blank squares represent correlations of *p* > 0.05. Tyrosol (TYR). Hydroxytyrosol (HT). Sum of TYR and HT (total simple phenols). Alkylresorcinols (ARs). Sum of AR-C17:0, AR-C19:0, AR-C21:0, AR-C23:0, AR-C25:0 (total AR). Xanthohumol (XN). Isoxanthohumol (IX). 8-prenylnaringenin (8PN). 6-prenylnaringenin (6PN). Sum of XN, IX, 8PN and 6PN (total prenylated flavonoids). Percentage of alcohol by volume (%ABV). International bitterness units (IBU).

Figure 1 represents the correlation matrix between all analyzed compounds, alcoholic content and beer bitterness of all beers. Total prenylated flavonoids and total simple phenols exhibited a moderate correlation with beer’s alcoholic content (*p* < 0.001) with correlation coefficients of 0.53 and 0.62, respectively. Beer bitterness (IBUs) presented a modest correlation with total prenylated flavonoids with a coefficient of 0.36. Weaker or non-significant correlations were observed among the three families of phenolic compounds analyzed. Significant correlations were obtained between compounds belonging to the same family.

### 2.6. Beer Styles

**Figure 2:**
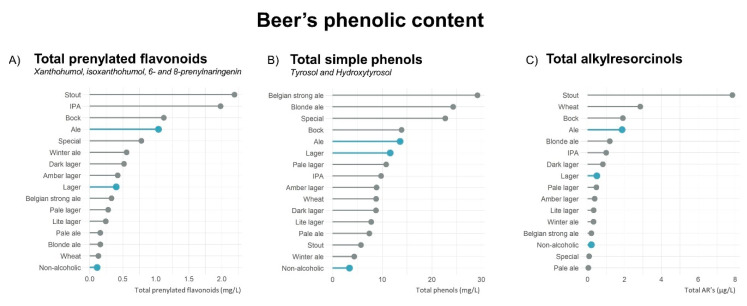
Beer’s phenolic content (**A**) total prenylated flavonoids, (**B**) total simple phenols and (**C**) total alkylresorcinols distributed by beer type (blue) in ale (*n* = 18); lager (*n* = 22) and non-alcoholic (*n* = 5) and in beer styles (grey) in amber lager (*n* = 2); Belgian strong ale (*n* = 3); blonde ale (*n* = 2); bock (*n* = 1); dark lager (*n* = 1); IPA (*n* = 6); lite lager (*n* = 1); pale ale (*n* = 1); pale lager (*n* = 15); special lager (*n* = 2); stout (*n* = 2); wheat (*n* = 2) and winter ale (*n* = 1). Results are expressed as mean concentration in descending order.

Figure 2 outlines the different families of phenolic compound concentrations across the beer’s styles. The beer styles with the highest concentrations of prenylated flavonoids were stout and Indian Pale Ale (IPA) with a mean (SD) of total prenylated flavonoids of 2.19 (3.10) and 1.98 (3.68) mg/L, respectively. In terms of total phenols, Belgian strong and blonde ale presented the highest concentrations: 29.2 (14.3) and 24.3 and 28.4 mg/L, respectively. Finally, stout, with a 7.84 (4.52) µg/L, was the beer style with the highest total ARs content. No statistical analysis was performed due to the low number of samples within certain beer styles.

## 3. Discussion

The present work studied a broad group of beers to describe their content of three families of phenolic compounds that have been associated with a wide range of potential biological activities and protective health effects. Specifically, this study characterizes beer’s antioxidant composition, showing that it is an important dietary source of prenylated flavonoids and the simple phenols TYR and HT. Moreover, our study reports, for the first time, the presence of low amounts of ARs in beer.

A distinctive ingredient of beer is the hop flower *(Humulus lupulus L*), which is added during the brewing process for its preserving properties and for its organoleptic characteristics. Beer is considered a unique source of these prenylated flavonoids in the diet. Urinary IX is used as a unique and accurate biomarker of beer consumption [19], which is in agreement with our analysis, pointing out IX as the main prenylated flavonoid present in beer. The type of fermentation, classifying beer into ale or lager was not associated with the prenylated flavonoid concentrations. Prenylated flavonoids are of interest due to their display of antibacterial, anti-inflammatory, antioxidant and other biological effects [20]. In particular, the compound XN is being closely studied for its potential chemopreventive properties. In the case of IX, and especially 8PN, these compounds are characterized by their strong phytoestrogen activities [21,22].

Malt phenolic compounds represent the main source of bioactive substances found in beer [23]. The most abundant are phenolic acids, phenolic alcohol, phenolic amines, phenolic amino acids and finally α-acids and iso-α-acids [24]. In the present study, we have focused on the analysis of the phenolic alcohols TYR and HT. The presence of the simple phenol TYR in relatively high concentrations in beer has been previously reported [25,26,27]. We confirmed the presence of TYR in beer and, additionally, we described, for the first time, the presence of HT in certain beers. TYR and HT are not present in beer as raw components, they are formed during the fermentation process. TYR is produced as a product of tyrosine metabolism generated by yeast in the Ehrlich pathway. A minor part of the TYR formed can be later hydroxylated to give rise to HT [28]. Based on the concentrations observed, beer is a relevant source of TYR in the diet. TYR average content in beer is comparable to white wine. Nevertheless, certain beers exhibited TYR levels at the same range as red wine, considered a good source of TYR, whose concentrations have been reported to be between 20.5 and 44.5 mg/L [29]. The contribution of beer as a direct source of HT is negligible. Moreover, the presence of TYR in beer is relevant, since it has been demonstrated that dietary TYR is converted in humans into HT [30,31]. Both TYR supplementation and its biotransformation into HT are capable of triggering relevant beneficial effects on the cardiovascular system [30]. HT is considered one of the strongest dietary antioxidants, with anti-inflammatory, antiproliferative, antiplatelet and proapoptotic activities [32]. Therefore, beer would represent an indirect source of HT via TYR hydroxylation. Consequently, beer should also be considered a relevant source of TYR and HT, together with the traditional dietary sources of extra virgin olive oil and wine. 

ARs are a group of phenolic lipids that contain a resorcinol (a benzene ring with two hydroxyl groups in positions 1 and 3 and an odd-numbered alkyl chain in the range of 15–25 carbons at position 5). They are present in the outer part of certain grains and in the products produced from them [33,34]. They have been described in barley, wheat, rye, oats, rice and other cereal grains, and the relative abundance of the different homologues varies depending on the type of cereal. AR-C25:0, the most abundant AR found in beer, is (accordingly) the dominant AR homologue in barley [35]. ARs are being studied for their potential biological activities. They have shown antioxidant activity [36], protecting against LDL oxidation [37], and also improving glucose and cholesterol metabolism [38]. Nevertheless, it is important to contextualize the sources of ARs in the diet and to understand that, although we describe the presence of ARs in beer in trace amounts, their contribution to total AR dietary intake would be almost negligible. The intake of ARs in countries with a high consumption ranges between 12 and 18 mg/day [30]. Based on our results, a glass of an average beer of 330 mL (equivalent to one standard drink) could represent an intake of 0.3 µg of total ARs and, therefore, a minor contribution to the overall amount of ARs ingested.

Beer’s alcoholic content was positively correlated with prenylated flavonoid, TYR and HT concentration. It has been described that, during fermentation, the presence of phenols with antioxidant activity within the wort protect yeast viability against the stress generated by high levels of ethanol [39]. Therefore, a high concentration of prenylated flavonoids with their inherent antioxidant activity would contribute to yeast stability, enhancing the fermentation process and increasing alcohol content. TYR and HT are byproducts of this fermentation. Another fact confirming the importance of fermentation in the phenolic profile of beer is that the variety of yeast strain used for beer brewing is capable of triggering differences in the antioxidant activity and total phenolic composition of the beer produced [40,41]. In the case of ARs, their amount was not correlated with the alcoholic content of beer, nor with any of the analyzed groups of phenolic compounds. Given that ARs are biomarkers of whole grain intake [34], and that there are known differences in AR composition depending on the cereal type [33], the presence of ARs in beer most likely derives from the cereals used for the elaboration of the beer and is independent of other beer compounds.

Non-alcoholic beer’s popularity has risen due to a concern about alcohol abuse and its health consequences. The production of beer with a limited alcohol content can be achieved by two approaches: limiting the fermentation process, and hence the alcohol production, or by using physical methods to remove the alcohol at the end of brewing [42]. On one hand, the concentration of the prenylated flavonoid IX in non-alcoholic beer was borderline significantly lower than ale and lager beers. On the other hand, and in agreement with a previous study [26], non-alcoholic beers presented lower TYR and lower HT content. TYR and HT are produced as byproducts of fermentation and its limitation during the dealcoholizing process is likely to have a negative effect on their accumulation levels. In non-alcoholic beer production, physical methods including thermal processes or inverse osmosis, are often used. These processes could trigger the degradation or the loss of IX, TYR and HT, explaining the lower concentrations observed in non-alcoholic beers. Our results suggest that the non-alcoholic brewing process has a detrimental impact on the content of the simple phenols and IX. In the case of ARs, no statistical differences were observed in the values present in non-alcoholic beer, suggesting the stability of these compounds during dealcoholizing. Finally, in the context of non-alcoholic beer consumption, it is worth to mention the role that has been attributed to alcohol in promoting the bioavailability of phenolic compounds. This has been recently demonstrated with a reduced bioavailability of TYR following non-alcoholic beer consumption [31]. Therefore, non-alcoholic beers, which have a low concentration of phenolic compounds from the outset and an absence of alcohol, would represent a minor source of phenolic compounds. 

Eventually, Figure 2 represents an exploratory overview of the concentrations of the analyzed phenolic compounds across different beer styles. In the case of total prenylated flavonoids, stout and IPA styles presented the greatest concentrations in this family. In the case of TYR and HT, Belgian strong and blonde ales exhibited the highest concentrations. Finally, stout beer stands out for its AR content. However, caution must be applied as certain beer styles were under-represented, with a low number of samples being available. Further studies should analyze a larger sample of beers belonging to the mentioned styles to confirm their high concentration of phenolic compounds. Research on the characteristics of the mentioned beer styles was performed to understand the reason behind the high concentrations of selected phenolic compounds. In the case of IPA beers, originally, this beer style was characterized with the greatest proportion of hops, as it is known for its antimicrobial properties that enhance beer stability. Therefore, a high proportion of prenylated flavonoids would be expected. In the case of Stout beers, a distinctive characteristic is the use of roasted barley as a starting material. Based on the high concentration of ARs, this step could facilitate the extraction of ARs from the cereal to the wort during the brewing process. Finally, Belgian strong ale, the beer with the highest concentration in TYR and HT, uses a specific and traditional yeast that could produce higher proportions of TYR [43]. Overall, these observations confirm the importance of the starting materials and the fermentation on the final concentration of phenolic compounds. 

Finally, it is important to mention that, despite the interesting beer antioxidant profiles described in the present paper and in the literature, it is important to highlight the importance of a moderate consumption of beer in the context of a healthy dietary pattern, such as the Mediterranean diet [23]. Excessive beer consumption can lead to an excessive body weight, hamper pancreatic function and increase the risk of cancer due to its ethanol content and also due to the low levels of toxic compounds [17].

Our study presents some strengths and limitations. A key strength of the present study is the high number of beers analyzed, including different beer types and styles. The quantitative assessment of three different families of phenolic compounds provides a broad perspective of the phenolic profile of beer. More specifically, we show that TYR, formed during the fermentation process, is a phenolic compound abundantly present in beer. Additionally, prenylated flavonoids that derive from the variety of hops used during the brewing process are present in lower amounts than TYR. Finally, ARs, which most likely come from the malted cereals selected as ingredients for brewing, are only present in trace amounts in beers. However, our analysis was limited by the fact that certain beer styles were under-represented, due to their low availability on the market. Our current research has only quantified three families of phenolic compounds; however, beer is an extremely complex drink with several phenolic compounds whose concentrations have not yet been assessed. 

Overall, the exploratory nature of the present research offers some insight into the phenolic composition of beer, highlighting it as an important dietary source of prenylated flavonoids, TYR and, indirectly, HT. Additionally, it extends our knowledge of the levels of phenolic compounds present in different beer styles, different beer types and non-alcoholic beers. This work represents a starting point in understanding beer’s antioxidant profile; however, future studies should assess the bioavailability and the potential synergies of the mentioned compounds in the context of moderate beer consumption and its potential health effects.

## 4. Materials and Methods 

### 4.1. Chemicals and Reagents

TYR, HT, 3-(4-hydroxyphenyl)-1-propanol, XN, IX and 8PN taxifolin and ammonium fluoride were purchased from Sigma-Aldrich (St. Louis, MO, USA). HT-D_3_, were purchased from Toronto Research Chemicals Inc. (Toronto, ON, Canada). 5-heptadecylresorcinol (AR C17:0), 5-nonadecylresorcinol (AR C19:0), 5-nonadecylresorcinol-D4 (AR C19:0-D_4_), 5-heneicosylresorcinol (AR C21:0), 5-tricosylresorcinol (AR C23:0), 5-pentacosylresorcinol (AR C25:0) were purchased from ReseaChem GmbH (Burgdorf, Switzerland). Methanol and acetonitrile were obtained from Merck (Darmstadt, Germany). Ultra-pure water was supplied by a Milli-Q^®^ purification system (Darmstadt, Germany). 

### 4.2. Samples and Sample Preparation

A total of 45 different beers were selected for the analysis of phenolic compounds. A 10 mL sample of each beer was stored in a Falcon tube at −20 °C until the analysis. Beer foam was removed from all samples by means of ultrasonication prior to any analysis. All determinations were performed in duplicate.

### 4.3. Extraction and Analysis of Prenylated Flavonoids: IX, XN, 8PN and 6PN

All the samples were filtered through a 0.45 mm polytetrafluoroethylene filter and 600 ng/mL of taxifolin was added as internal standard. Aliquots of 10 µL were injected into the liquid chromatography coupled to mass spectrometry (LC–MS/MS) system without any other pretreatment. The identification and quantification of IX, XN, 6PN, and 8PN was performed using an Acquity UHPLC^®^ system equipped with a Waters binary pump system (Waters, Milford, MA, USA) coupled to an API 3000™ triple quadrupole mass spectrometer (Sciex, Concord, ON, Canada) with a turbo ion spray source working in a negative mode. Chromatographic separation was performed with a Luna C18^®^ column, 50 mm × 2.0 mm i.d., 5 mm (Phenomenex, Torrance, CA, United States), using 5 mM ammonium bicarbonate buffer adjusted to pH 7.0 as an aqueous mobile phase and acetonitrile and methanol (1:1 proportion) as an organic phase. For the quantification of analytes, the multiple reaction monitoring (MRM) mode was used, monitoring 3 transitions: 353–119 (IX and XN), 339–219 (8PN and 6PN), and 303–285 (taxifolin) [19]. Calibration curves were prepared adding standards to pure water.

### 4.4. Extraction and Analysis of Simple Phenols TYR and HT in Beer

TYR and HT content were determined by LC–MS/MS following a dilute-and-shoot approach. Samples were diluted 40 times with a mobile phase (65% A: 35% B) and spiked with 10 µL of an internal standard containing 1 µg/mL of 3-(4-hydroxyphenyl)-1-propanol and 1 µg/mL of HT-D_3_. Mobile phase A contained 0.5 mM of ammonium fluoride in water. Mobile phase B contained 0.5 mM of ammonium fluoride in methanol. All samples were analyzed by LC–MS/MS (Agilent Technologies, Santa Clara, CA, USA). The separation was carried out with an Acquity UPLC^®^ BEH C18 column 1.7μm particle size, 3 mm × 100 mm (Waters, Milford, MA, USA). The following transitions were monitored on the acquisition method in MRM mode: 137–106 (TYR), 151–106 (3-(4-hydroxyphenyl)-1-propanol), 153–123 (HT) and 156–126 (HT-D_3_). Calibration curves were prepared adding standards of TYR and HT to pure water.

### 4.5. Extraction and Analysis of ARs in Beer

Extraction of AR from beers was based on a liquid-liquid extraction protocol using ethyl acetate as described for the analysis of ARs in cereals [39]. Briefly, 1 mL of each beer was spiked with 20 µL of internal standard (AR C19:0D_4_ at 50 ng/mL). ARs were extracted using 4 mL of ethyl acetate for 24 h. Then, the organic layer was evaporated to dryness under nitrogen (40 °C and 15 psi) and reconstituted in 0.25 mL of methanol. Then, it was centrifuged for 5 min at 4 °C (12.000 rpm) to obtain a clear supernatant fraction, which was directly injected into the LC–MS/MS system. Chromatographic separation of five ARs was performed by using an Acquity UPLC^®^ instrument (Waters, Milford, MA, USA) operated using MassLynx 4.1 software. The LC system was equipped with an Acquity UPLC^®^ (BEH C18 1.7 µm 2.1 × 100 mm) column (Waters, Milford, MA, USA). The injection volume was 10 µL, the flow rate was 0.3 mL/min, and the temperature of the column was set at 55 °C. An isocratic method was selected with a solution of 0.5 mM ammonium fluoride in methanol as a mobile phase solvent. The detection was performed with a triple quadrupole mass spectrometer (Xevo^®^ TQS-Micro MS, Waters, Milford, MA, USA) equipped with an orthogonal Z-spray-electrospray ionization source (ESI). The monitoring and quantification of AR was performed in MRM mode, monitoring the following transitions: 347–305 (AR C17:0), 375–122 (AR 19:0), 403–361 (AR 21:0), 431–389 (AR 23:0), 459–417 (AR 25:0), and 379–337 (AR 19:0-D_4_). Calibration curves were prepared by adding standards to pure water.

### 4.6. Statistical Analysis

Statistical analyses and figures were performed using the R software (R Foundation for Statistical Computing, Vienna, Austria), version 3.5.2. The normality of continuous variables was assessed by normal probability plots and non-parametric tests were used if data did not follow a normal distribution. The R packages used were ‘corrplot’ and ‘tidyverse’. The significance level was set at *p* < 0.05.

## Figures and Tables

**Table 1:** Beer phenolic composition of prenylated flavonoids, simple phenols tyrosol (TYR) and hydroxytyrosol (HT) and alkylresorcinols (ARs) according to beer type (ale vs. lagers vs. free).

Compound	All Beers	Type			*p* ^a^	*p* ^b^	*p* ^c^
		Ale	Lager	Non-Alcoholic			
IX (mg/L)	0.34 (0.41)	0.42 (0.54)	0.33 (0.31)	0.08 (0.08)	0.76	0.06	0.06
XN (mg/L)	0.17 (0.87)	0.39 (1.38)	0.03 (0.04)	0.01 (0.02)	0.98	0.55	0.55
8PN (mg/L)	0.03 (0.10)	0.06 (0.15)	0.02 (0.03)	0.00 (0.00)	0.78	0.55	0.49
6PN (mg/L)	0.08 (0.34)	0.17 (0.53)	0.02 (0.03)	0.01 (0.01)	0.84	0.50	0.57
Total PN (mg/L)	0.62 (1.51)	1.04 (2.33)	0.40 (0.37)	0.11 (0.98)	0.80	0.17	0.17
TYR (mg/L)	11.45 (10.55)	13.53 (12.94)	11.58 (8.75)	3.38 (2.60)	0.87	**0.01**	**<0.01**
HT (mg/L)	0.03 (0.03)	0.04 (0.03)	0.02 (0.02)	0.01 (0.01)	**0.02**	**0.01**	**0.05**
Total simple phenols (mg/L)	11.5 (10.5)	13.6 (12.9)	11.6 (8.8)	3.4 (2.6)	0.90	**0.01**	**0.01**
AR-C17:0 (µg/L)	0.00 (0.01)	0.01 (0.02)	0.00 (0.00)	0.00 (0.00)	0.08	0.32	0.70
AR-C19:0 (µg/L)	0.07 (0.15)	0.13 (0.20)	0.03 (0.07)	0.00 (0.00)	0.44	0.44	0.44
AR-C21:0 (µg/L)	0.19 (0.40)	0.39 (0.55)	0.07 (0.18)	0.02 (0.03)	0.23	0.50	0.91
AR-C23:0 (µg/L)	0.17 (0.36)	0.30 (0.49)	0.09 (0.21)	0.03 (0.02)	0.27	0.45	0.68
AR-C25:0 (µg/L)	0.58 (1.19)	1.04 (1.69)	0.31 (0.59)	0.15 (0.11)	0.34	0.58	0.83
Total ARs (µg/L)	1.01 (2.03)	1.87 (2.84)	0.50 (1.04)	0.2 (0.15)	0.25	0.58	0.93

Results shown as mean (SD); *p* = *p*-value from Kruskal-Wallis test, comparing (a) ales vs. lagers; (b) non-alcoholic vs. ales; (c) non-alcoholic vs. lagers. Bold values denote statistical significance at the *p* < 0.05 level. Standard deviation (SD). Tyrosol (TYR). Hydroxytyrosol (HT). Alkylresorcinols (ARs). Sum of AR-C17:0, AR-C19:0, AR-C21:0, AR-C23:0, AR-C25:0 (total AR). Xanthohumol (XN). Isoxanthohumol (IX). 8-prenylnaringenin (8PN). 6-prenylnaringenin (6PN). Sum of XN, IX, 8PN and 6PN (total PN).

## Supplementary Materials

The following are available online, Table S1: List and characteristics of the analyzed beers.

## References

[1] World Health Organization (2018). Global Status Report on Alcohol and Health 2018.

[2] Poli A., Marangoni F., Avogaro A., Barba G., Bellentani S., Bucci M., Cambieri R., Catapano A.L., Costanzo S., Cricelli C. (2013). Moderate alcohol use and health: A consensus document. Nutr. Metab. Cardiovasc. Dis..

[3] Costanzo S., Di Castelnuovo A., Donati M.B., Iacoviello L., de Gaetano G. (2011). Wine, beer or spirit drinking in relation to fatal and non-fatal cardiovascular events: A meta-analysis. Eur. J. Epidemiol.

[4] de Gaetano G., Costanzo S., Di Castelnuovo A., Badimon L., Bejko D., Alkerwi A. (2016). Effects of moderate beer consumption on health and disease: A consensus document. Nutr. Metab Cardiovasc Dis.

[5] Chiva-Blanch G., Arranz S., Lamuela-Raventos R.M., Estruch R. (2013). Effects of wine, alcohol and polyphenols on cardiovascular disease risk factors: Evidences from human studies. Alcohol Alcoholism.

[6] Jerkovic V., Callemien D., Collin S. (2005). Determination of stilbenes in hop pellets from different cultivars. J. Agric Food Chem.

[7] Wannenmacher J., Gastl M., Becker T. (2018). Phenolic Substances in Beer: Structural Diversity, Reactive Potential and Relevance for Brewing Process and Beer Quality. Compr. Rev. Food Sci. Food Saf..

[8] Neveu V., Perez-Jiménez J., Vos F., Crespy V., du Chaffaut L., Mennen L. (2010). Phenol-Explorer: An online comprehensive database on polyphenol contents in foods. Database (Oxford).

[9] Gerhäuser C. (2005). Beer constituents as potential cancer chemopreventive agents. Eur. J. Cancer.

[10] Nardini M., Natella F., Scaccini C., Ghiselli A. (2006). Phenolic acids from beer are absorbed and extensively metabolized in humans. J. Nutr. Biochem..

[11] Piazzon A., Forte M., Nardini M. (2010). Characterization of phenolics content and antioxidant activity of different beer types. J. Agric. Food Chem..

[12] Floridi S., Montanari L., Marconi O., Fantozzi P. (2003). Determination of free phenolic acids in wort and beer by coulometric array detection. J. Agric. Food Chem..

[13] Řehová L., Škeříkova V., Jandera P. (2004). Optimisation of gradient HPLC analysis of phenolic compounds and flavonoids in beer using a CoulArray detector. J. Sep. Sci..

[14] Jandera P., Škeříková V., Řehová L., Hájek T., Baldriánová L., Škopová G. (2005). RP-HPLC analysis of phenolic compounds and flavonoids in beverages and plant extracts using a CoulArray detector. J. Sep. Sci..

[15] Nardini M., Garaguso I. (2020). Characterization of bioactive compounds and antioxidant activity of fruit beers. Food Chem..

[16] Quifer-Rada P., Vallverdú-Queralt A., Martínez-Huélamo M., Chiva-Blanch G., Jáuregui O., Estruch R. (2015). A comprehensive characterisation of beer polyphenols by high resolution mass spectrometry (LC-ESI-LTQ-Orbitrap-MS). Food Chem..

[17] Osorio-Paz I., Brunauer R., Alavez S. (2019). Beer and its non-alcoholic compounds in health and disease. Crit. Rev. Food Sci. Nutr..

[18] Hernandes K.C., Souza-Silva É.A., Assumpção C.F., Zini C.A., Welke J.E. (2019). Validation of an analytical method using HS-SPME-GC/MS-SIM to assess the exposure risk to carbonyl compounds and furan derivatives through beer consumption. Food Addit. Contam. Part. A.

[19] Quifer-Rada P., Martínez-Huélamo M., Chiva-Blanch G., Jáuregui O., Estruch R., Lamuela-Raventós R.M. (2014). Urinary Isoxanthohumol Is a Specific and Accurate Biomarker of Beer Consumption. J. Nutr..

[20] Chen X., Mukwaya E., Wong M.-S., Zhang Y. (2014). A systematic review on biological activities of prenylated flavonoids. Pharm Biol.

[21] Stevens J.F., Page J.E. (2004). Xanthohumol and related prenylflavonoids from hops and beer: To your good health!. Phytochemistry.

[22] Štulíková K., Karabín M., Nešpor J., Dostálek P. (2018). Therapeutic perspectives of 8-prenylnaringenin, a potent phytoestrogen from hops. Molecules.

[23] Arranz S., Chiva-Blanch G., Valderas-Martínez P., Medina-Remón A., Lamuela-Raventós R.M., Estruch R. (2012). Wine, beer, alcohol and polyphenols on cardiovascular disease and cancer. Nutrients.

[24] Quesada-Molina M., Muñoz-Garach A., Tinahones F.J., Moreno-Indias I. (2019). A New Perspective on the Health Benefits of Moderate Beer Consumption: Involvement of the Gut Microbiota. Metabolites.

[25] Li M., Yang Z., Hao J., Shan L., Dong J. (2008). Determination of Tyrosol, 2-Phenethyl Alcohol, and Tryptophol in Beer by High-Performance Liquid Chromatography. J. Am. Soc. Brew. Chem..

[26] Cerrato-Alvarez M., Bernalte E., Bernalte-García M.J., Pinilla-Gil E. (2019). Fast and direct amperometric analysis of polyphenols in beers using tyrosinase-modified screen-printed gold nanoparticles biosensors. Talanta.

[27] Bartolomé B., Peña-Neira A., Gómez-Cordovés C. (2000). Phenolics and related substances in alcohol-free beers. Eur. Food Res. Technol..

[28] Hazelwood L.A., Daran J.M., Van Maris A.J.A., Pronk J.T., Dickinson J.R. (2008). The Ehrlich pathway for fusel alcohol production: A century of research on Saccharomyces cerevisiae metabolism. Appl. Environ. Microbiol..

[29] Piñeiro Z., Cantos-Villar E., Palma M., Puertas B. (2011). Direct liquid chromatography method for the simultaneous quantification of hydroxytyrosol and tyrosol in red wines. J. Agric. Food Chem..

[30] Boronat A., Mateus J., Soldevila-Domenech N., Guerra M., Rodríguez-Morató J., Varon C. (2019). Cardiovascular benefits of tyrosol and its endogenous conversion into hydroxytyrosol in humans. A randomized, controlled trial. Free Radic. Biol. Med..

[31] Soldevila-Domenech N., Boronat A., Mateus J., Diaz-Pellicer P., Matilla I., Pérez-Otero M. (2019). Generation of the Antioxidant Hydroxytyrosol from Tyrosol Present in Beer and Red Wine in a Randomized Clinical Trial. Nutrients.

[32] Robles-Almazan M., Pulido-Moran M., Moreno-Fernandez J., Ramirez-Tortosa C., Rodriguez-Garcia C., Quiles J.L. (2018). Hydroxytyrosol: Bioavailability, toxicity, and clinical applications. Food Res. Int..

[33] Landberg R., Marklund M., Kamal-Eldin A., Åman P. (2014). An update on alkylresorcinols–Occurrence, bioavailability, bioactivity and utility as biomarkers. J. Funct. Foods.

[34] Rodríguez-Morató J., Jayawardene S., Huang N.K., Dolnikowski G.G., Galluccio J., Lichtenstein A.H. (2020). Simplified method for the measurement of plasma alkylresorcinols: Biomarkers of whole-grain intake. Rapid Commun. Mass Spectrom..

[35] Chen Y., Ross A.B., Åman P., Kamal-Eldin A. (2004). Alkylresorcinols as Markers of Whole Grain Wheat and Rye in Cereal Products. J. Agric. Food Chem..

[36] Wang Z., Hao Y., Wang Y., Liu J., Yuan X., Sun B. (2019). Wheat alkylresorcinols protect human retinal pigment epithelial cells against H2O2-induced oxidative damage through Akt-dependent Nrf2/HO-1 signaling. Food Funct..

[37] Parikka K., Rowland I.R., Welch R., Wähälä K. (2006). In vitro antioxidant activity and antigenotoxicity of 5-n-alkylresorcinols. J. Agric. Food Chem..

[38] Oishi K., Yamamoto S., Itoh N., Nakao R., Yasumoto Y., Tanaka K. (2015). Wheat alkylresorcinols suppress high-fat, high-sucrose diet-induced obesity and glucose intolerance by increasing insulin sensitivity and cholesterol excretion in male mice. J. Nutr..

[39] Gharwalova L., Sigler K., Dolezalova J., Masak J., Rezanka T., Kolouchova I. (2017). Resveratrol suppresses ethanol stress in winery and bottom brewery yeast by affecting superoxide dismutase, lipid peroxidation and fatty acid profile. World J. Microbiol. Biotechnol..

[40] Capece A., Romaniello R., Pietrafesa A., Siesto G., Pietrafesa R., Zambuto M. (2018). Use of Saccharomyces cerevisiae var. boulardii in co-fermentations with S. cerevisiae for the production of craft beers with potential healthy value-added. Int. J. Food Microbiol..

[41] Basso R.F., Alcarde A.R., Portugal C.B. (2016). Could non-Saccharomyces yeasts contribute on innovative brewing fermentations?. Food Res. Int..

[42] Ross A.B. (2012). Analysis of Alkylresorcinols in Cereal Grains and Products Using Ultrahigh-Pressure Liquid Chromatography with Fluorescence, Ultraviolet, and CoulArray Electrochemical Detection. J. Agric. Food Chem..

[43] Strong G., England K. (2015). Guide of Styles 2015 Beer Judge Certification Program. https://www.bjcp.org/stylecenter.php.

