# Supplementary material for: Beer Phenolic Composition of Simple Phenols, Prenylated Flavonoids and Alkylresorcinols
Source: Molecules. 2020 Jun 2;25(11):2582. doi: 10.3390/molecules25112582 (PMC7321207; doi:10.3390/molecules25112582)
Supplement: Supplementary file 1 [file molecules-25-02582-s001.pdf]

molecules

MDPI

# Supporting information

**Table S1.** List and characteristics of the analyzed beers ($n$ = 45).

| Type | Commercial Name | Style | ABV (%) | IBU | Country |
|---|---|---|---|---|---|
| Ale ($n$ = 18) | Leffe Radieuse | Belgian strong ale | 8,2 | 25 | Belgium |
| | Delirium Tremens | Belgian strong ale | 8,5 | 26 | Belgium |
| | Judas | Belgian strong ale | 8,5 | 25 | Belgium |
| | Leffe Blonde | Blonde ale | 6.6 | 20 | Belgium |
| | Espiga Blonde Ale | Blonde ale | 4,5 | 25 | Spain |
| | Damm complot IPA | IPA | 6.6 | 47 | Spain |
| | Moritz Red IPA | IPA | 5,4 | 50 | Spain |
| | Espiga Black IPA | IPA | 8,5 | 78 | Spain |
| | IPA Montseny | IPA | 6,4 | 48 | Spain |
| | Montseny Lupulus | IPA | 5,4 | 29 | Spain |
| | Vic Ale IPA | IPA | 6,2 | 50 | Spain |
| | Espiga Pale Ale | Pale ale | 5,0 | 30 | Spain |
| | Guinness | Stout | 4,3 | 45 | Ireland |
| | Montseny Negra | Stout | 5,2 | 35 | Spain |
| | Hoegaarden | Wheat | 4,9 | 15 | Belgium |
| | Paulaner | Wheat | 5,5 | 12 | Germany |
| | Franziskaner | Wheat | 5,0 | 12 | Germany |
| | Socarrada | Winter ale | 5,0 | 24 | Spain |
| Non-alcoholic ($n$ = 5) | Free Damm | | 0,0 | 17 | Spain |
| | Ambar 0,0 | | 0,0 | 17 | Spain |
| | Moritz 0,0 | | 0,0 | 17 | Spain |
| | Heineken 0,0 | | 0,0 | 18 | Netherlands |
| | San Miguel 0,0 | | 0,0 | 17 | Spain |
| Lager ($n$ = 22) | San Miguel Selecta | Amber lager | 6.2 | 32 | Spain |
| | Voll Damm | Amber lager | 7.2 | 34 | Spain |
| | Bock damm | Bock | 5.9 | 25 | Spain |
| | Mahou Negra | Dark lager | 5.5 | 25 | Spain |
| | Saaz Damm | Lite lager | 3.5 | 10.5 | Spain |
| | Alhambra 1925 | Pale lager | 6.4 | 25 | Spain |
| | Mahou Maestra | Pale lager | 7.5 | 28 | Spain |

| | | | | |
|---|---|---|---|---|
| Xibeca | Pale lager | 4.6 | 18 | Spain |
| Moritz | Pale lager | 5.4 | 21 | Spain |
| Estrella Damm | Pale lager | 5.4 | 12 | Spain |
| Brutus | Pale lager | 6.0 | 23 | Spain |
| Stella Artois | Pale lager | 5.2 | 24 | Belgium |
| Alhambra Especial | Pale lager | 5,4 | 17 | Spain |
| A.K. Damm | Pale lager | 4,8 | 13 | Spain |
| Estrella Galicia | Pale lager | 5,5 | 24 | Spain |
| Heineken | Pale lager | 5,0 | 16 | Netherlands |
| Pilsner Urquell | Pale lager | 4,4 | 40 | Czech Republic |
| Corona | Pale lager | 4,5 | 19 | Mexico |
| Radeberger | Pale lager | 4,8 | 33 | Germany |
| Schwaben Bräu Das Helle | Pale lager | 5,0 | 15 | Germany |
| Cruzcampo Gran Reserva | Special | 6,4 | 25 | Spain |
| Malquerida | Special | 5,0 | 19 | Spain |

%ABV = percentage of alcohol by volume. IBU = International bitterness units.
